# Effects of Vitamin D Supplementation on Fatigue and Disease Activity in Systemic Lupus Erythematosus

**DOI:** 10.7759/cureus.78830

**Published:** 2025-02-10

**Authors:** Oana Raluca Predescu, Florentin Ananu Vreju, Anca Emanuela Musetescu, Alesandra Florescu, Stefan Cristian Dinescu, Cristina Elena Bita, Andreea Lili Barbulescu, Paulina Lucia Ciurea

**Affiliations:** 1 Doctoral School, University of Medicine and Pharmacy of Craiova, Craiova, ROU; 2 Department of Rheumatology, University of Medicine and Pharmacy of Craiova, Craiova, ROU; 3 Department of Pharmacology, University of Medicine and Pharmacy of Craiova, Craiova, ROU

**Keywords:** disease activity, fatigue, supplementation, systemic lupus erythematosus, vitamin d

## Abstract

Background: Systemic lupus erythematosus (SLE) is an autoimmune disease characterized by chronic inflammation and various clinical symptoms, with vitamin D deficiency suggested as a contributing factor. This study aimed to evaluate the effects of vitamin D supplementation on fatigue and disease activity in SLE patients.

Methods: Patients diagnosed based on EULAR/ACR 2019 criteria were divided into three groups: no supplementation, 4000 IU, and 8000 IU of vitamin D daily for six months. Clinical assessments included serum complement levels (C3 and C4), fatigue scores (Functional Assessment of Chronic Illness Therapy (FACIT)-Fatigue and Fatigue Severity Scale (FSS)), and disease activity (Safety of Estrogens in Lupus Erythematosus National Assessment-Systemic Lupus Erythematosus Disease Activity Index (SELENA-SLEDAI)).

Results: Results showed significant increases in vitamin D levels and serum complement levels in the supplementation groups. Serum complement levels and fatigue scores improved significantly in both the 4000 IU and 8000 IU groups. Additionally, there was a slight reduction in SELENA-SLEDAI scores in the treated groups, but without statistical significance.

Conclusions: The findings suggest that vitamin D supplementation positively affects fatigue and some parameters of disease activity in SLE patients, though its overall impact on disease activity needs further investigation.

## Introduction

Systemic lupus erythematosus (SLE) is a complex autoimmune disorder marked by inflammation that affects the human immune system and manifests with a broad spectrum of symptoms. It is a multifaceted autoimmune illness characterized by various immunological abnormalities [1,2]. These include abnormal production of autoantibodies, impaired removal of immune complexes and apoptotic bodies by phagocytes, and inadequate modulation of B-cell function by T-cells [3,4].

The exact pathophysiology of SLE remains incompletely understood; however, it is linked to genetic, environmental, hormonal, and immunological factors. Vitamin D deficiency is considered a significant contributor to the pathogenesis of SLE not only from an immunological but also from an environmental point of view [5,6]. 

In the last decade, the discovery of the expression of vitamin D receptors in immune system cells has spurred further investigation into the immune-modulating effects of vitamin D. Both the innate and adaptive immune systems comprise many types of cells, including macrophages, dendritic cells, T lymphocytes, and B-cells, which exhibit this kind of receptors [7]. These have the ability to react to the physiologically active form of vitamin D, known as 1,25-dihydroxyvitamin D. Multiple studies conducted worldwide have consistently found a higher prevalence of vitamin D insufficiency among individuals with lupus compared to healthy ones. One possible reason for this is that individuals with SLE tend to avoid the sun, as it is a known promoter for lupus exacerbation [8,9].

Multiple globally conducted studies have found a greater prevalence of deficient serum levels of vitamin D in lupus patients [10]. Consequently, 87% of SLE patients were found to have low levels of vitamin D, whereas 48% of these patients were determined to have insufficient levels of vitamin D [11]. The data in the literature regarding vitamin D levels and SLE disease activity are contrasting. Even if vitamin D insufficiency has been associated with disease flares, there is strong disagreement regarding the correlation between disease activity and serum vitamin D levels [12]. Some studies have demonstrated a negative correlation, while others have not found an association at all [13,14].

Some authors note that low vitamin D levels may be considered a possible risk factor for SLE, rather than an effect of the disease itself. The majority of these studies have suggested that in order to implement the aforementioned recommendations, it is mandatory to ascertain the therapeutic effectiveness of vitamin D supplementation in this specific patient group or population at risk [15]. Several interventional clinical trials have reviewed the effects of administering vitamin D supplements on disease activity, as well as on additional complications like fatigue or proteinuria [16].

Studies conducted on experimental mice models of lupus have shown a notable enhancement in the skin-related symptoms of the disease, reduction in proteinuria, and higher rates of survival in lupus patients receiving vitamin D supplementation [17]. The study's objective was to identify the effects of vitamin D supplementation in SLE individuals on serum complement levels, fatigue scores, and disease activity.

## Materials and methods

Patients

Patients diagnosed with systemic lupus erythematosus according to the European Alliance of Associations for Rheumatology (EULAR)/American College of Rheumatology (ACR) 2019 criteria [18] were included in the study. The individuals were hospitalized in the Department of Rheumatology of the Emergency Clinical County Hospital of Craiova between October 2023 and December 2023. The study received approval from the local institutional ethics committee of the University of Medicine and Pharmacy of Craiova (Registration No. 205/20.09.2023), according to the European Union Guidelines (Declaration of Helsinki). Informed consent was obtained from all of the patients.

The following requirements had to be met in order to be enrolled in the study: age over 18 years, SLE diagnosis, prednisone intake less than 15 mg daily, insufficient or deficient levels of 25-hydroxy (OH) vitamin D, and no current or prior to six months vitamin D supplementation. The main exclusion criteria were the coexistence of an overlap syndrome or other autoimmune disorders, trauma, severe infections, liver disease, stage IV and V kidney disease, pregnancy, and breastfeeding.

Demographic features and evaluation of clinical and laboratory data

Physical examination and the collection of blood tests were performed on all patients. Blood samples were collected in specific tubes for each test (ethylenediaminetetraacetic acid (EDTA): lavender top for complete blood count (CBC) and erythrocyte sedimentation rate (ESR) and serum separator tubes; and gold top for liver enzymes, serum creatinine, C3 and C4, vitamin D, and antibodies). The tubes were labeled with patient information, date, and time and transported to the laboratory, following appropriate guidelines for temperature and handling to maintain sample integrity. 

The clinical assessment included evaluation of the cutaneous involvement and specific joint testing for pain and swelling. The patients’ fatigue and disease activity were assessed using the Functional Assessment of Chronic Illness Therapy-Fatigue Scale (FACIT-Fatigue) [19], the Fatigue Severity Scale (FSS) [20], and the Safety of Estrogens in Lupus Erythematosus National Assessment-Systemic Lupus Erythematosus Disease Activity Index (SELENA-SLEDAI) [21].

The following serum parameters were assessed: CBC, ESR, liver enzymes, serum creatinine, and C3 and C4 fractions of the serum complement with normal values of 90-180 mg/dl and 10-40 mg/dl, respectively. Values of 25(OH) vitamin D less than or equal to 20 ng/ml were considered a deficiency, while levels between 21 and 29 ng/ml were considered an insufficiency. Vitamin D levels were determined using the electrochemiluminescence (ECLIA) method. Also, the patients were tested for antibody positivity as follows: anti-dsDNA, anti-Smith, anti-Ro, anti-La, anti-ribosomal P protein, and anti-nucleosome antibodies. 

Treatment and outcome

The patients were divided 1:1:1 into three groups. The first group received no vitamin D supplementation, the second group received 4000 IU of vitamin D in the form of cholecalciferol, and the third group received 8000 IU of cholecalciferol daily for six months. Serum levels of C3, C4, 25(OH) vitamin D, FACIT-Fatigue, FSS, and SELENA-SLEDAI were measured not only at baseline but also at six months.

Statistical analysis

Statistical analysis was conducted using GraphPad Prism 10.2.3 (GraphPad Software, USA) for Windows. Statistics between variables were examined using the paired t-test and Pearson or Spearman correlation coefficients. One-way analysis of variance (ANOVA) was employed to assess differences between groups. A p-value of <0.05 was taken into consideration as statistically significant. Mean ± standard deviation (SD) is depicted for continuous variables. A post hoc power analysis was performed for the ANOVA test with three groups. With a total sample size of 60 patients and a significance level of 0.05, the calculated power was 0.78 for detecting a large effect size (f = 0.40). 

## Results

Baseline characteristics of the three groups

We screened 114 patients diagnosed with SLE and divided 60 patients into three groups (1:1:1). The female-to-male ratio was 54:6. The mean age of individuals included in the study was 43.23 ± 12.65 years. 

In the untreated group, according to SELENA-SLEDAI, 20 (100%) had a mild/moderate flare. In the 4000 IU group, the disease activity was distributed as follows: mild/moderate flare, 18 (90%); and severe flare, two (10%). In the 8000 IU group, 19 (95%) and one (5%) presented mild/moderate and severe flare, respectively.

In the untreated group, nine (45%) individuals had low serum levels of C3 complement, while 10 (50%) presented C4 hypocomplementemia. In the 4000 IU group, 12 (60%) patients presented with C3 hypocomplementemia, while 11 (55%) had low C4 levels. In the 8000 IU group, 11 (55%) and seven (35%) patients had low C3 and C4 levels, respectively.

In the untreated group, 10 (50%) patients had insufficient levels of vitamin D, while 10 (50%) were deficient. In the 4000 IU and 8000 IU groups, deficiency was encountered in nine (45%) patients in both groups. Also, insufficient levels of vitamin D were found in 11 (55%) patients in both groups. The baseline characteristics of the groups are presented in Table 1.

**Table 1:** Baseline characteristics of the study groups SELENA-SLEDAI: Safety of Estrogens in Lupus Erythematosus National Assessment-Systemic Lupus Erythematosus Disease Activity Index; FACIT-Fatigue: Functional Assessment of Chronic Illness Therapy (FACIT)-Fatigue; FSS: Fatigue Severity Scale

Characteristics	Untreated (n = 20)	4000 IU (n = 20)	8000 IU (n = 20)	p-value
Sex (females) (n, %)	18 (90%)	18 (90%)	18 (90%)	
Age (years) (mean ± SD)	45.82 ± 12.78	43.81 ± 12.56	40.06 ± 12.62	0.576
Duration of disease (years) (mean ± SD)	9.3 ± 3.48	9.7 ± 4.21	9.83 ± 3.97	0.431
C3 (mg/dL) (mean ± SD)	88.27 ± 8.84	86.05 ± 9.48	86.78 ± 8.40	0.724
C4 (mg/dL) (mean ± SD)	10.61 ± 4.31	10.85 ± 4.54	12.47 ± 4.98	0.391
Vitamin D levels (ng/mL) (mean ± SD)	20.24 ± 4.41	20.43 ± 6.52	21.16 ± 4.91	0.848
SELENA-SLEDAI (mean ± SD)	11.85 ± 0.58	12.1 ± 0.30	12.05 ± 0.22	0.126
FACIT-Fatigue (mean ± SD)	24.3 ± 3.07	25.4 ± 3.16	25.7 ± 2.12	0.266
FSS (mean ± SD)	47.6 ± 1.31	47.75 ± 1.37	47.9 ± 1.77	0.819

The clinical manifestations of the patients and the administered treatment are presented in Table 2. 

**Table 2:** Clinical manifestations and treatment of the study groups

Manifestations (n, %)	Untreated (n = 20)	4000 IU (n = 20)	8000 IU (n = 20)	
Cutaneous	17 (85%)	18 (90%)	17 (85%)	
Articular	12 (60%)	10 (50%)	14 (70%)	
Renal	3 (15%)	3 (15%)	5 (25%)	
Hematological	4 (20%)	5 (25%)	2 (10%)	
Serositis	2 (10%)	1 (5%)	0 (0%)	
Neurological	1 (5%)	0 (0%)	1 (5%)	
Treatment (n, %)				
Hydroxychloroquine	20 (100%)	20 (100%)	20 (100%)	
Azathioprine	3 (15%)	5 (25%)	4 (20%)	
Mycophenolate mofetil	5 (25%)	3 (15%)	5 (25%)	
Cyclophosphamide	1 (5%)	0 (0%)	1 (5%)	
Glucocorticoids				
<5 mg daily	2 (10%)	3 (15%)	4 (20%)	
5-10 mg daily	17 (85%)	14 (70%)	13 (65%)	
10-15 mg daily	1 (5%)	3 (15%)	3 (15%)	

The positivity for the tested autoantibodies is presented in Table 3.

**Table 3:** Antibody positivity in the study group

Antibody	Untreated (n = 20)	4000 IU (n = 20)	8000 IU (n = 20)
Anti-dsDNA (n, %)	15 (75%)	10 (50%)	15 (75%)
Anti-Smith (n, %)	1 (5%)	5 (25%)	3 (15%)
Anti-Ro (n, %)	1 (5%)	2 (10%)	0 (0%)
Anti-La (n, %)	0 (0%)	0 (0%)	0 (0%)
Anti-ribosomal P protein (n, %)	1 (5%)	2 (10%)	1 (5%)
Anti-nucleosome (n, %)	2 (10%)	1 (5%)	1 (5%)

Comparison of the group characteristics between baseline and six months

The baseline and six-month characteristics of the groups are presented in Tables 4-6.

**Table 4:** Baseline and 6 months characteristics of the untreated group SELENA-SLEDAI: Safety of Estrogens in Lupus Erythematosus National Assessment-Systemic Lupus Erythematosus Disease Activity Index; FACIT-Fatigue: Functional Assessment of Chronic Illness Therapy (FACIT)-Fatigue; FSS: Fatigue Severity Scale

Untreated group	Baseline	6 months	p-value
C3 (mg/dL)(mean, SD)	88.27 (8.84)	88.51 (8.91)	0.084
C4 (mg/dL) (mean, SD)	10.61 (4.31)	10.60 (4.32)	0.933
Vitamin D levels (mg/dL) (mean, SD)	20.24 (4.41)	20.24 (4.51)	0.960
FACIT-Fatigue (mean, SD)	24.3 (3.07)	24.3 (2.99)	0.313
FSS (mean, SD)	47.6 (1.31)	47.75 (1.48)	0.831
SELENA-SLEDAI (mean, SD)	11.85 (0.58)	11.7 (1.41)	0.667

**Table 5:** Baseline and six-month characteristics of the 4000 IU group SELENA-SLEDAI: Safety of Estrogens in Lupus Erythematosus National Assessment-Systemic Lupus Erythematosus Disease Activity Index; FACIT-Fatigue: Functional Assessment of Chronic Illness Therapy (FACIT)-Fatigue; FSS: Fatigue Severity Scale

4000 IU group	Baseline	6 months	p-value
C3 (mg/dL)(mean, SD)	86.05 (9.48)	105.32 (2.88)	<0.001
C4 (mg/dL) (mean, SD)	10.85 (4.54)	23.60 (3.63)	<0.0001
Vitamin D levels (mg/dL) (mean, SD)	20.43 (6.52)	33.80 (10.99)	<0.0001
FACIT-Fatigue (mean, SD)	25.4 (3.16)	33.0 (3.47)	<0.0001
FSS (mean, SD)	47.75 (1.37)	41.85 (1.69)	<0.0001
SELENA-SLEDAI (mean, SD)	12.1 (0.30)	11.35 (2.08)	0.104

**Table 6:** Baseline and six-month characteristics in the 8000 IU group SELENA-SLEDAI: Safety of Estrogens in Lupus Erythematosus National Assessment-Systemic Lupus Erythematosus Disease Activity Index; FACIT-Fatigue: Functional Assessment of Chronic Illness Therapy (FACIT)-Fatigue; FSS: Fatigue Severity Scale

8000 IU group	Baseline	6 months	p-value
C3 (mg/dL)(mean, SD)	86.78 (8.40)	107.14 (3.31)	<0.0001
C4 (mg/dL) (mean, SD)	12.47 (4.98)	23.38 (3.08)	<0.001
Vitamin D levels (mg/dL) (mean, SD)	21.16 (4.91)	34.27 (8.52)	<0.0001
FACIT-Fatigue (mean, SD)	25.7 (2.12)	38.5 (2.80)	<0.0001
FSS (mean, SD)	47.9 (1.77)	38.15 (1.78)	<0.0001
SELENA-SLEDAI (mean, SD)	12.05 (0.22)	10.95 (2.50)	0.069

**Figure 1:**
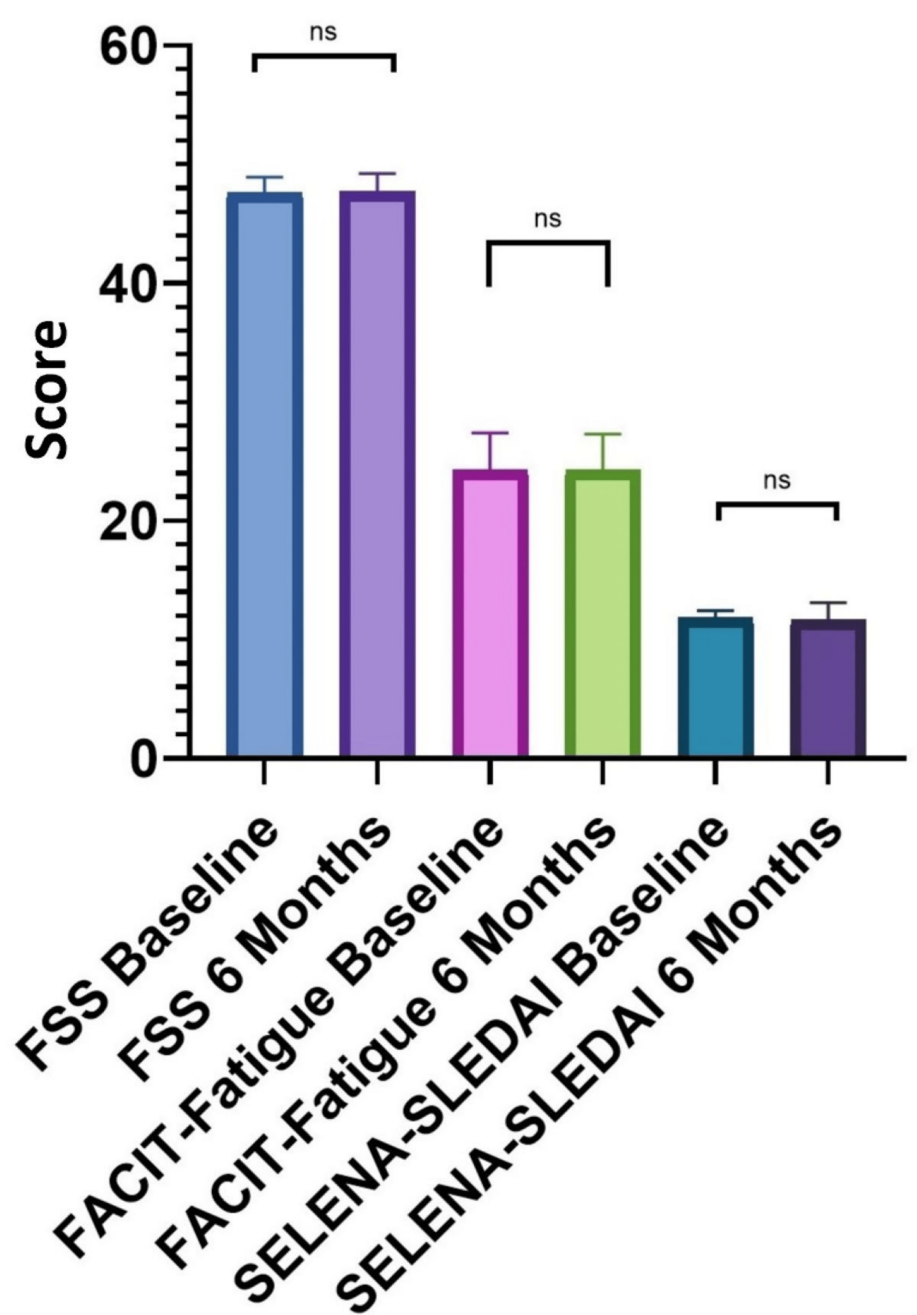
FACIT-Fatigue, FSS, and SELENA-SLEDAI comparison at baseline and at six months in the untreated group SELENA-SLEDAI: Safety of Estrogens in Lupus Erythematosus National Assessment-Systemic Lupus Erythematosus Disease Activity Index; FACIT-Fatigue: Functional Assessment of Chronic Illness Therapy (FACIT)-Fatigue; FSS: Fatigue Severity Scale

There were no statistically significant differences between the baseline and six-month measurements for the untreated group. Neither the serum complements nor the levels of vitamin D increased statistically significantly without treatment. Also, the fatigue scores and disease activity scores at six months were comparable to those at baseline (Figure 1).

**Figure 2:**
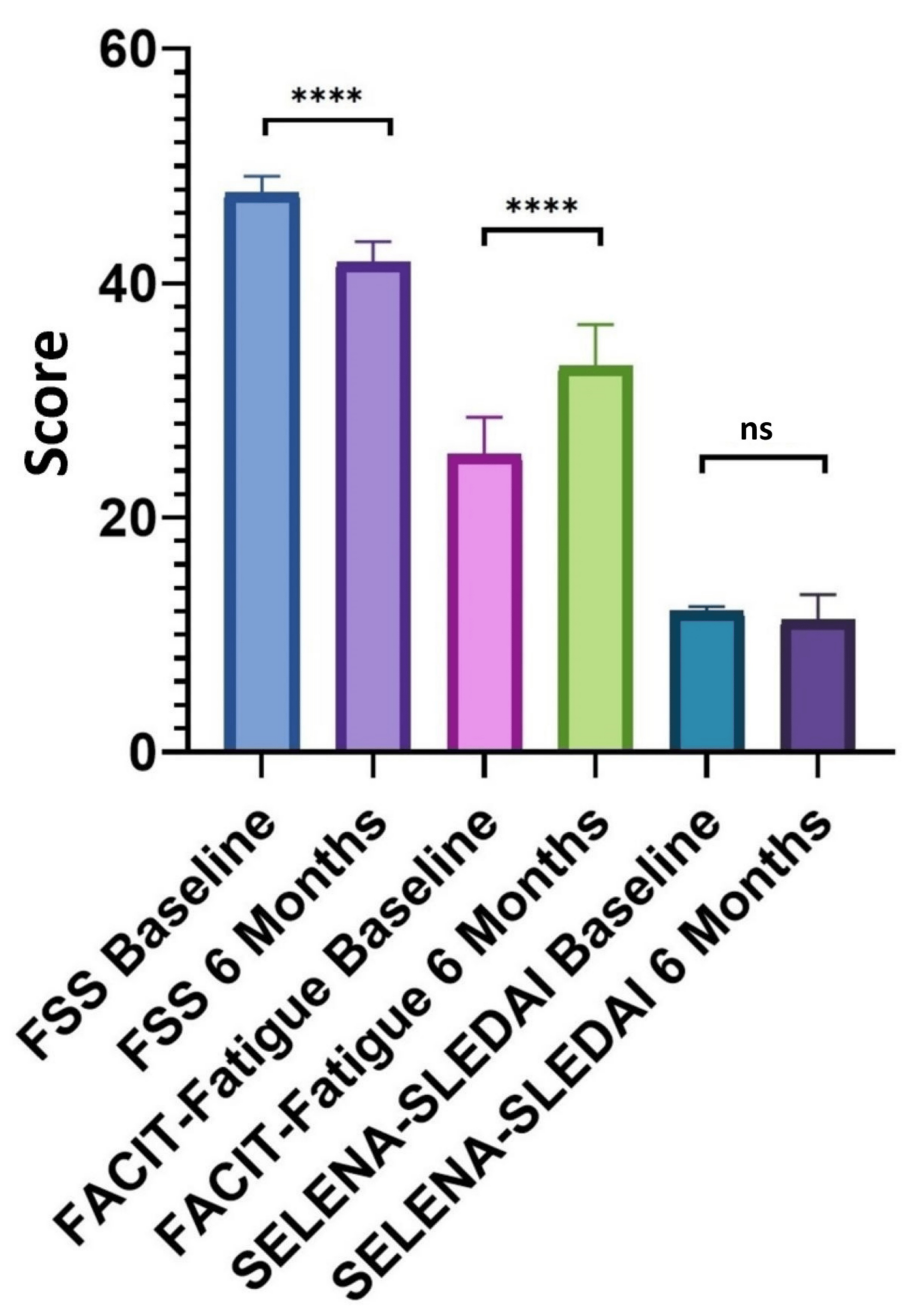
FACIT-Fatigue, FSS, and SELENA-SLEDAI comparison at baseline and at six months in the 4000 IU group SELENA-SLEDAI: Safety of Estrogens in Lupus Erythematosus National Assessment-Systemic Lupus Erythematosus Disease Activity Index; FACIT-Fatigue: Functional Assessment of Chronic Illness Therapy (FACIT)-Fatigue; FSS: Fatigue Severity Scale

In the 4000 IU group, the mean serum levels of C3 registered a significant increase of 22.4% (p < 0.001) from a statistical point of view, while C4 registered an increase of 117.44% (p < 0.0001). Vitamin D levels increased by 65.43% (p < 0.0001) at six months compared to baseline values. FACIT-Fatigue and FSS scores have a statistically significant improvement of 29.92% (p < 0.0001) and 12.36% (p < 0.0001), respectively. SELENA-SLEDAI registered a 6.2% decrease after six months of supplements, but it was not statistically significant (p = 0.104) (Figure 2).

**Figure 3:**
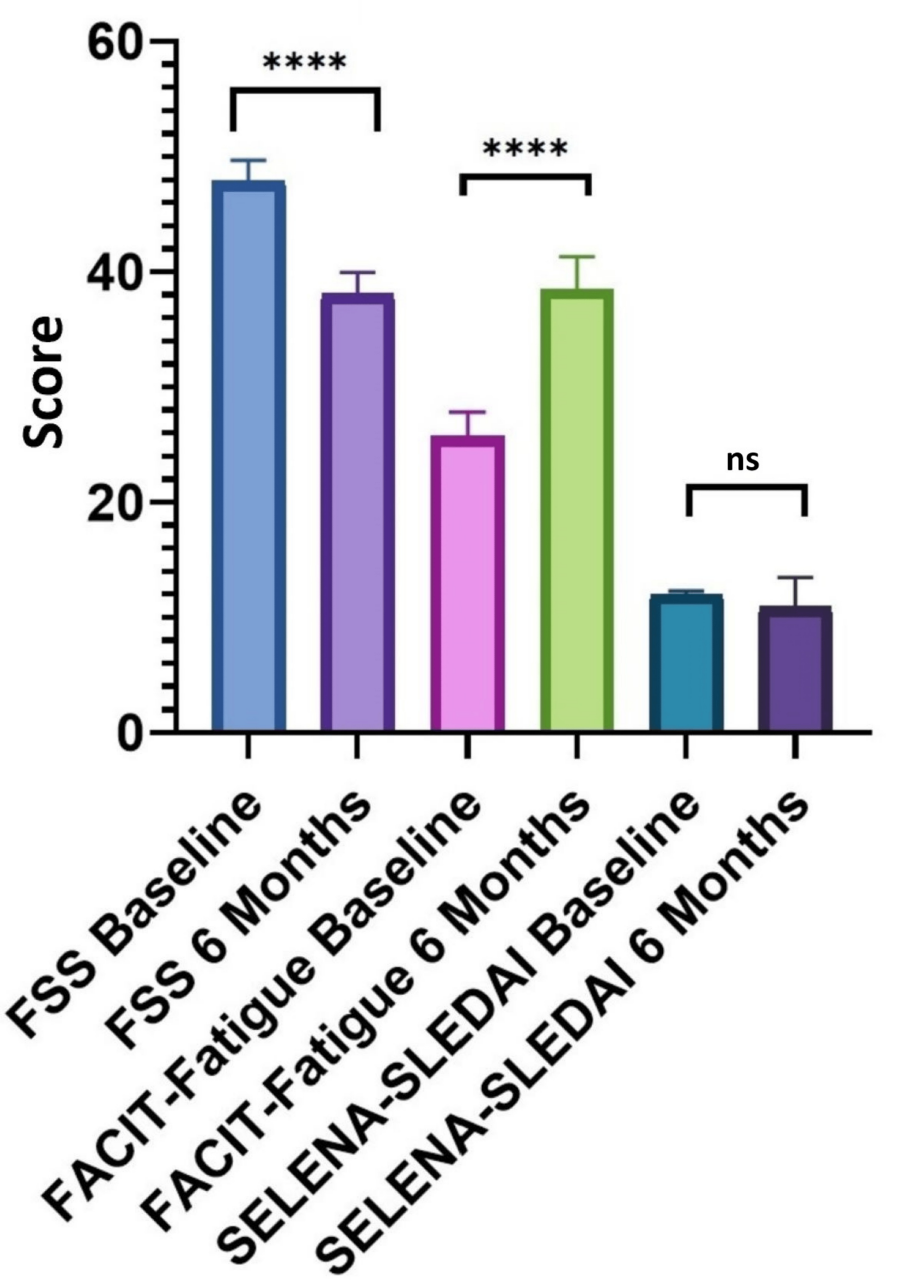
FACIT-Fatigue, FSS, and SELENA-SLEDAI comparison at baseline and at six months in the 8000 IU group SELENA-SLEDAI: Safety of Estrogens in Lupus Erythematosus National Assessment-Systemic Lupus Erythematosus Disease Activity Index; FACIT-Fatigue: Functional Assessment of Chronic Illness Therapy (FACIT)-Fatigue; FSS: Fatigue Severity Scale

In the 8000 IU group, the mean serum levels of C3 and C4 registered a statistically significant increase of 23.46% (p < 0.0001) and 87.55% (p < 0.001 ) from baseline to six months, respectively. The increase in mean vitamin D levels was 61.95% (p < 0.001). Regarding fatigue, the mean FACIT-Fatigue and FSS scores improved by 49.81% (p < 0.0001) and 20.35% (p < 0.0001), respectively. The disease activity score SELENA-SLEDAI registered a nonsignificant improvement of 9.13% from a statistical point of view (p = 0.069) (Figure 3).

Comparison at six months between the three groups

The mean values and SD of the study groups at six months are presented in Table 7.

**Table 7:** Mean values of vitamin D, FACIT-Fatigue, FSS, and SELENA-SLEDAI at six months in the three groups SELENA-SLEDAI: Safety of Estrogens in Lupus Erythematosus National Assessment-Systemic Lupus Erythematosus Disease Activity Index; FACIT-Fatigue: Functional Assessment of Chronic Illness Therapy (FACIT)-Fatigue; FSS: Fatigue Severity Scale

6 months	Untreated	4000 IU	8000 IU	p-value
C3	88.51 (8.91)	105.32 (2.88)	107.14 (3.31)	<0.001
C4	10.60 (4.32)	23.60 (3.63)	23.38 (3.08)	<0.001
Vitamin D levels (mg/dL) (mean, SD)	20.24 (4.51)	33.80 (10.99)	34.27 (8.52)	<0.0001
FACIT-Fatigue (mean, SD)	24.3 (2.99)	33.0 (3.47)	38.5 (2.80)	<0.0001
FSS (mean, SD)	47.75 (1.48)	41.85 (1.69)	38.15 (1.78)	<0.0001
SELENA-SLEDAI (mean, SD)	11.7 (1.41)	11.35 (2.08)	10.95 (2.50)	0.515

Vitamin D levels (p < 0.0001), FACIT-Fatigue (p < 0.0001), and FSS (p < 0.0001) showed significant differences from a statistical point of view between the untreated and treated groups. Although the disease activity score improved from baseline in the groups treated with 4000 IU and 8000 IU of vitamin D, SELENA-SLEDAI did not show a statistically significant difference (p = 0.515) when comparing the three groups at six months, indicating that the treatment did not have an important impact on disease severity as measured by this index.

Associations between vitamin D and disease activity

The levels of vitamin D at baseline and six months in the untreated group showed no association with SELENA-SLEDAI, p = 0.633 and p = 0.614, respectively (Table 8). Also, in the group treated with 4000 IU daily cholecalciferol, the levels of vitamin D both at baseline and after six months were not associated with the disease activity score, with p-values being 0.771 and 0.053 in the group who received 8000 IU daily of vitamin D, and serum levels of 25(OH) vitamin D were not associated with SELENA-SLEDAI with p-values of 0.680 and 0.693, respectively. 

**Table 8:** Associations between SELENA-SLEDAI and vitamin D levels at baseline and six months SELENA-SLEDAI: Safety of Estrogens in Lupus Erythematosus National Assessment-Systemic Lupus Erythematosus Disease Activity Index

SLEDAI-vitamin D association	Untreated group	4000 IU group	8000 IU group
Baseline	p = 0.633	p = 0.771	p = 0.680
6 months	p = 0.614	p = 0.053	p = 0.693

## Discussion

This study analyzed the possible effects of vitamin D supplementation on disease activity and fatigue in patients with lupus. The findings of our research demonstrate a positive impact on various important factors, such as vitamin D levels, FACIT-Fatigue scores, FSS scores, and SELENA-SLEDAI score, in the groups that were administered 4000 IU and 8000 IU of cholecalciferol.

The study addresses a controversial subject in the current literature, providing new insights into the potential immunological benefits of vitamin D supplementation in SLE management. Also, the research highlights the real-world relevance by conducting the study in a clinical setting with a diverse patient population.

These findings align with prior studies in the field, including the study conducted by Karimzadeh et al., which has its primary purpose as the assessment of the effectiveness of vitamin D supplements on disease activity in SLE patients with vitamin D deficiency. The trial included 45 patients who were randomized into two groups. The first group, known as the interventional group, received a weekly dose of 50,000 vitamin D units for 12 weeks, followed by a dose of 50,000 units per month for three months. The second group received a placebo. The levels of vitamin D and SLEDAI scores were examined both at baseline and after supplementation. The interventional group showed a considerable rise in vitamin D levels, from an average of 17.36 ± 4.26 ng/ml to 37.69 ± 5.92 ng/ml (p < 0.001), in contrast to the placebo group. Also, there was no difference from a statistical point of view in disease activity (SLEDAI) when comparing the two groups both before and after the supplementation [22].

Ruiz-Irastorza et al. showed that vitamin D supplementation effectively elevates serum vitamin D levels in lupus patients. In this study, 60 patients received a daily dose of 800 IU of vitamin D over the course of 12 months. The results revealed a relevant augmentation in 25(OH) vitamin D levels in all the patients, most notable in those who had lower serum levels prior to supplementation. Fatigue measured using the Visual Analogue Scale showed a negative association with 25(OH) vitamin D levels. However, no statistically significant associations were proven between disease activity scores measured by SLEDAI or Systemic Damage Index (SDI) and the increases in vitamin D levels [23]. 

Aranow et al. performed a study on 57 SLE individuals who were randomized into three groups. The first group received a placebo, and the second and third received 2000 IU and 4000 IU of vitamin D daily for a period of 12 weeks. However, the study did not prove a statistically significant difference in disease activity scores measured using SELENA-SLEDAI among the three groups, similar to the findings in our study [24].

In 2016, Achmad Rifa’i et al. developed a study on 39 SLE individuals who were further split into a placebo group and a group who was administered 1200 IU of vitamin D daily. The authors showed a statistically significant elevation in serum vitamin D levels in the treated group in comparison with the placebo. Similar to our findings, a statistically significant decrease in SLEDAI and FSS was noted. Also, the authors proved a negative association between vitamin D levels and SLEDAI prior to and after supplementation with vitamin D [25].

A study by Lima et al. in 2016 highlighted the effects of vitamin D supplements on managing juvenile-onset SLE. This randomized, double-blinded, placebo-controlled trial included 40 patients with juvenile-onset SLE who were randomized into two groups: one placebo group and the other receiving 50000 IU of cholecalciferol weekly over a period of 24 weeks. The authors demonstrated that the group who had been administered vitamin D supplementation had notably higher serum levels of 25(OH) vitamin D compared with the placebo group. Regarding disease activity, the treated group showed a statistically significant decrease in both SLEDAI and the European Consensus Lupus Activity Measurement (ECLAM). Additionally, there was a noteworthy reduction in fatigue in the vitamin D group, findings similar to our study [26].

Our study failed to show a significant difference from a statistical point of view between the groups regarding SELENA-SLEDAI at six months, findings similar to a randomized, double-blinded, placebo-controlled study by Pakchotanon et al. in 2020. The authors aimed to ascertain the safety and effectiveness of ergocalciferol in SLE patients. The patients were divided into two groups: one received a placebo, while the other group received 100000 IU per week for four weeks, followed by 40000 IU weekly for 20 weeks. Only 88 individuals completed the evaluation at baseline and 24 weeks. Serum vitamin D levels were significantly more elevated in the ergocalciferol group, while no significant difference was noted between the two groups, from a statistical point of view, regarding SLEDAI-2K scores, flare events, ESR, and CRP levels. However, one of the highlights of the study was the fact that a larger number of patients in the ergocalciferol group managed to reduce the glucocorticoid doses both at 12 and 24 weeks [27].

In 2020, a cross-sectional study by Rodriguez et al. investigated the effect of dietary intake and supplementation of vitamin D in a group of 285 SLE patients. The study showed that neither clinical nor laboratory parameters were affected by vitamin D intake levels. The only notable difference was related to serum C3 levels which were significantly more elevated in the individuals who were administered vitamin D supplements. These findings suggest that vitamin D supplementation might positively influence SLE activity, specifically by increasing serum complement C3 levels [28].

A prospective open-label study by Magro et al. in 2021, conducted on 31 individuals with either vitamin D deficiency or insufficiency, evaluated the effects of vitamin D supplementation on disease activity, fatigue, and the expression of the interferon signature gene in patients with SLE. They were given vitamin D3 supplements: 8000 IU per day for eight weeks for individuals with deficiency, or four weeks for those with insufficiency, followed by a maintenance dose of 2000 IU daily for 12 months. The results showed a decrease in disease activity as calculated using SLEDAI-2K (p = 0.028) and a reduction in fatigue severity (p = 0.071) at 12 months, findings comparable to our study [29].

In a comparative, observational, cross-sectional study by Garcia-Carrasco in 2016 which evaluated 137 SLE patients, the authors found comparable vitamin D levels among the patients in remission and those with disease flares. Additionally, there was no relation between disease activity scores and vitamin D levels, findings consistent with our study [14].

Nevertheless, the evidence in the literature is contrasting. Some studies show an inverse or negative association between disease activity scores and vitamin D levels, while others prove no association. A study by Yap et al. proved that low vitamin D levels were associated with high disease activity in SLE patients [13].

In a meta-analysis by Zheng et al., the authors concluded that vitamin D supplementation does not have significant effects on disease activity in lupus patients. Regardless, they also pointed out a slight tendency to decrease disease activity after supplementation with vitamin D, without statistically significant differences, results comparable to our findings [30]. 

Our study shows the beneficial effects of vitamin D supplementation in SLE patients. However, several limitations should be acknowledged, including the small sample size, the absence of a cohort study design, and the single-center setting, which may introduce biases related to the study population. To validate the findings of this study, further multicenter, randomized, double-blinded, placebo-controlled trials with a larger sample size and extended follow-up period are needed.

## Conclusions

The objective of the study was to ascertain the impact of vitamin D supplementation on fatigue and disease activity in individuals diagnosed with systemic lupus erythematosus. The findings suggest that taking vitamin D supplements, at doses of both 4000 IU and 8000 IU per day, significantly enhances the serum levels of vitamin D, as well as the complement levels, and reduces fatigue as measured by FACIT-Fatigue and FSS scores. Moreover, the groups who received vitamin D supplementation experienced a slight decrease in disease activity scores (SELENA-SLEDAI), but it was not statistically significant. Nevertheless, there were no notable disparities in disease activity between the treated groups and the untreated group at six months.

The results indicate that administering vitamin D supplements can successfully decrease fatigue while improving specific immunological factors in SLE individuals. However, additional research is needed to determine its influence on the overall activity of the disease. Based on the observed potential benefits, it is worth considering the inclusion of vitamin D supplementation as a component of the treatment approach for managing symptoms associated with SLE, namely, fatigue. Subsequent investigations should prioritize conducting extensive, multicenter studies with extended periods of observation to confirm these findings and investigate the longstanding advantages and safety of administering high-dose vitamin D supplements to individuals with systemic lupus erythematosus.
